# A representation and deep learning model for annotating ubiquitylation sentences stating E3 ligase - substrate interaction

**DOI:** 10.1186/s12859-021-04435-7

**Published:** 2021-10-18

**Authors:** Mengqi Luo, Zhongyan Li, Shangfu Li, Tzong-Yi Lee

**Affiliations:** 1 Warshel Institute for Computational Biology, The Chinese University of Hong Kong, Shenzhen, China; 2 School of Life Sciences, University of Science and Technology of China, Hefei, China; 3 School of Life and Health Sciences, The Chinese University of Hong Kong, Shenzhen, China

**Keywords:** Ubiquitylation sentences annotation, Text mining, Deep learning, E3 ligase, Natural language processing, Information representation

## Abstract

**Background:**

Ubiquitylation is an important post-translational modification of proteins that not only plays a central role in cellular coding, but is also closely associated with the development of a variety of diseases. The specific selection of substrate by ligase E3 is the key in ubiquitylation. As various high-throughput analytical techniques continue to be applied to the study of ubiquitylation, a large amount of ubiquitylation site data, and records of E3-substrate interactions continue to be generated. Biomedical literature is an important vehicle for information on E3-substrate interactions in ubiquitylation and related new discoveries, as well as an important channel for researchers to obtain such up to date data. The continuous explosion of ubiquitylation related literature poses a great challenge to researchers in acquiring and analyzing the information. Therefore, automatic annotation of these E3-substrate interaction sentences from the available literature is urgently needed.

**Results:**

In this research, we proposed a model based on representation and attention mechanism based deep learning methods, to automatic annotate E3-substrate interaction sentences in biomedical literature. Focusing on the sentences with E3 protein inside, we applied several natural language processing methods and a Long Short-Term Memory (LSTM)-based deep learning classifier to train the model. Experimental results had proved the effectiveness of our proposed model. And also, the proposed attention mechanism deep learning method outperforms other statistical machine learning methods. We also created a manual corpus of E3-substrate interaction sentences, in which the E3 proteins and substrate proteins are also labeled, in order to construct our model. The corpus and model proposed by our research are definitely able to be very useful and valuable resource for advancement of ubiquitylation-related research.

**Conclusion:**

Having the entire manual corpus of E3-substrate interaction sentences readily available in electronic form will greatly facilitate subsequent text mining and machine learning analyses. Automatic annotating ubiquitylation sentences stating E3 ligase-substrate interaction is significantly benefited from semantic representation and deep learning. The model enables rapid information accessing and can assist in further screening of key ubiquitylation ligase substrates for in-depth studies.

## Background

Ubiquitylation is one kind of an important post-translational modification of protein that plays central role in cellular coding. The biological functions of ubiquitylation cover a wide range, such as proteasomal degradation, assembly of multiprotein compounds, intracellular transportation, inflammatory signaling, autophagy, regulation of DeoxyriboNucleic Acid (DNA) repair, enzymatic activity and so on [1]. Ubiquitylation is also closely linked to the development of multiple diseases such as Alzheimer’s disease, Parkinson’s disease, multiple cancers and so on [2]. Regulation of ubiquitylation on the organism is related to the specific selectivity of E3 ligase for substrates. Since ubiquitylation ligase E3 interacts directly with the substrate, its contribution to the specificity and selectivity of the substrate protein can not to be neglected [3]. Currently, over 600 E3 ligases with functional annotations have been identified. The E3-substrate interaction can help to understand the pathogenesis of ubiquitylation-related diseases, and develop drugs with high specificity and low side effects [4].

As various high-throughput analytical techniques continue to be applied to the study of protein ubiquitylation, a large amount of data on ubiquitylation sites, records of E3-substrate interaction continue to be produced. Biomedical literature provides new way to record, publish, store and transmit information of E3-substrate interaction in ubiquitylation, and they are usually an important vehicle for related new discoveries and an important channel for researchers to obtain such up to date data. The biomedical literatures on ubiquitylation contain evidentiary depictions of E1 activating enzymes, E2 ubiquitylation-conjugating enzymes, E3 ligases, substrates and ubiquitylation sites in their cascade enzymatic reactions [5–9]. And also, databases have been created based on these literatures, for instance, database named (The Ubiquitin and Ubiquitin-like Conjugation Database) UUCD [10] collected data of E1, E2, E3 ligase and deubiquitylating enzyme (DUB) deubiquitylating enzymes from scientific literatures, as well as provided their functional annotation and classification. However, with the rapid development of the biomedical field in recent years, the amount of relationship between ubiquitylation sites, E3 ligase and substrate interactions presents explosive growth, as a consequence, the continuous explosion of the corresponding research literature has posed a great challenge to researchers and database administrators in accessing and analyzing the information. Automatic extraction and annotation of these E3-substrate interaction sentences from biomedical literature can facilitate researchers and database administrators in acquiring, organizing, summarizing and analyzing the corresponding information, so as to assist in the related research and exploration in this field. Hence, we propose this research, which aims to construct a model for automatic annotating E3-substrate interaction sentences from literature. Our contributions are two folds:

First, we created a manual corpus for E3-substrate interaction sentences, which provides positive sentences and negative sentences as well as their E3 ligases and (or) substrates annotated;

Second, we proposed an attention mechanism based deep learning model, which exploits representation and cooperates with Long Short-Term Memory (LSTM) deep learning method, to automatic annotate E3-substrate interaction sentences in biomedical literature.

Up to now, almost all of the names of E3 ligases are known, while the names of many substrates are still in the exploration stage. A study showed that in recent years, for human proteins as example in mUbiSiDa (version 1.0), only about 15% of 5700 substrates have the known corresponding e3 ligase [2]. And, given the names of a pair of e3 and substrates, it is also unknown whether there is a ubiquitylation relationship between them. Thus, we manually created a E3-substrate interaction sentences corpus, which consists of sentences all containing E3 protein, and these sentences may narrate the E3-substrate interaction or not. The aim of our model is to classify sentences containing E3 protein(s) into positive class or negative class. Our model construction has two stages: first, by applying natural language processing techniques, we extract significant features from sentences and transform them into multiple dimension vectors for representation; second, the representation vectors are input into an LSTM-based neural network for training. We also compared our attention mechanism based deep learning-based model with other statistical machine learning-based models. The experimental results proved that our proposed model not only can achieve well annotation performance, but also outperforms other statistical machine learning-based models.

## Methods

### Problem description

We aim to extract sentences that state particular relationship between e3 and substrate in ubiquitylation, in other words, e3 targets substrate, and both e3 and substrate are proteins. For examples, “*These data identify Mdm2 as a novel E3 ligase for FOXOs and extend the analogous mode of regulation between FOXO and p53*”, “*Mdm2*” is E3 ligase and “*FOXOs*” is substrate. Extracting these sentences from text means distinguish them from other sentences that not state this ubiquitylation relationship between e3 and substrate, thus, we identify the extraction task as a binary classification problem. First, analyze and implement representation for these sentences; second, train a model by exploiting attention mechanism based deep learning classifier. It is worth to mention that we only focus on classifying sentences with E3 proteins inside. The model is to learn a mapping function f: X $\to$ Y, which would predict label (positive or negative) for particular input sentence.

### Overview of the model

**Fig. 1:**
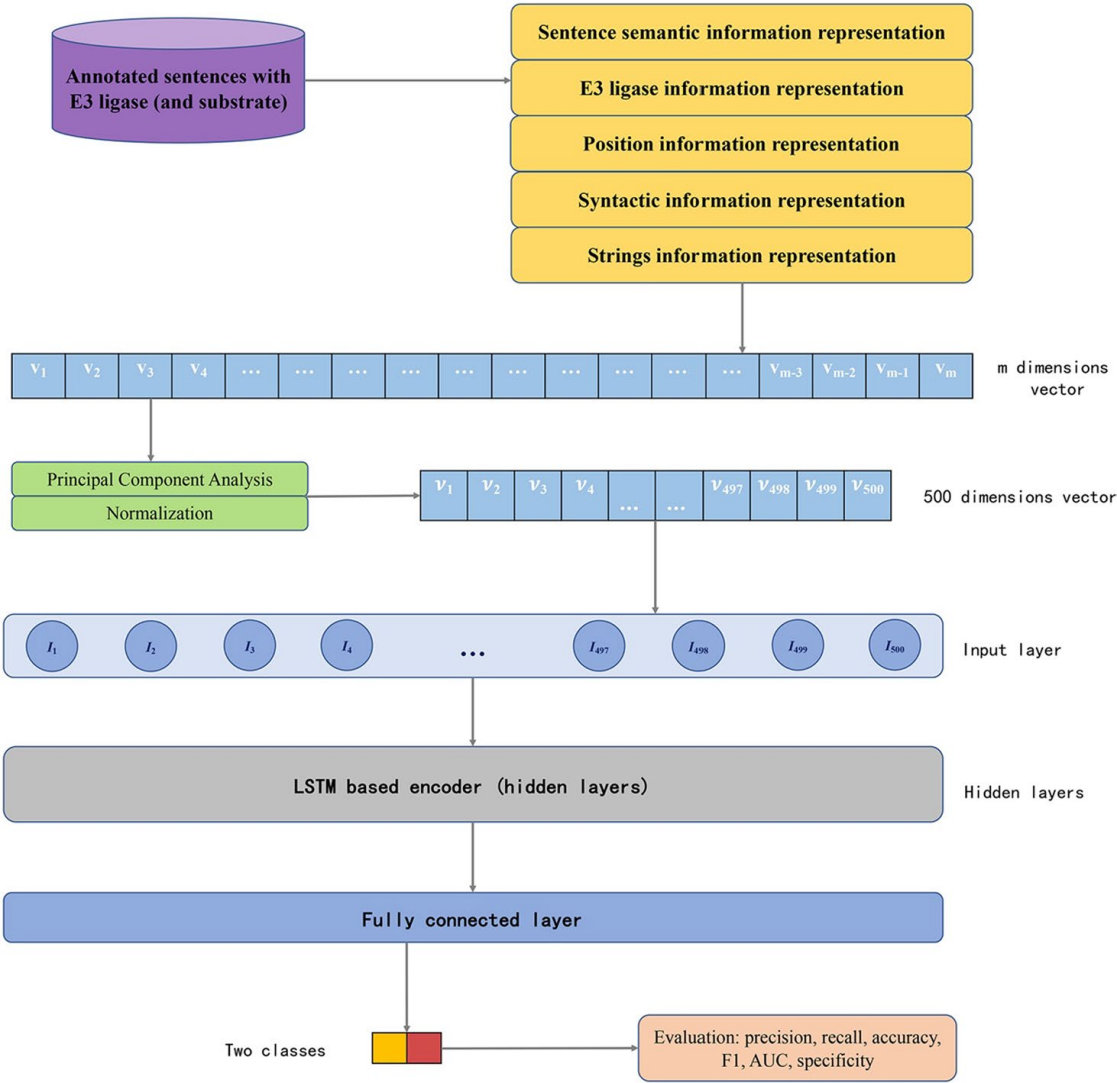
Model construction process. Five representation methods are applied for a E3-interaction sentence information, and they generated 5 vectors. Then Principal Component Analysis and Normalization are applied to reduce the dimension of each vector to 100, and finally a 500-dimension vector is formed. The 500-dimension vector is input into LSTM-based neural network for encoding, and the final encoding vector is input into the fully connected layer. The fully connected layer processes the vector and allocates class label for this sentence

Our model consists of two parts: information representation and deep learning-based classifier. In the first stage, natural language processing methods are applied to generate sentence semantic information representation, E3 ligase information representation, position information representation, syntactic based information representation and strings information representation, and obtains the multiple dimension vector; in the second stage, the representation vector is input into a LSTM based encoder, and the encoder generates a final encoding vector. At last, the final encoding is input into a fully connected layer to obtain output values and class labels. Figure 1 shows the whole model construction process.

### Input information representation

Input information to the deep learning classifier is represented by low dimension vectors. In our corpus (detailed in “Datasets” section), all selected sentences contain at least one E3 protein. Sentences stating at least one E3-substrate interaction were regarded as positive examples, and other sentences were regarded as negative examples. By applying natural language processing techniques, we exploited five aspects of information: sentence semantic, E3 semantic, syntactic, position and strings.

#### Sentence semantic information

The semantic and conceptual information of sentences are significant for model construction. Capture this information can deal with changing the order of words or the use of word variants or synonyms in different sentences with similar semantic information.

#### E3 ligase information

To exploit E3 information in our model.

#### Position information

As mentioned above, we used a dictionary of E3 for sentence selection in preprocessing of our model. The absolute position of E3 in sentence can provide significant information for extracting E3-substrate ubiquitylation sentence. We extract E3 position by detecting its ranking in all words of the sentence. For example, if one sentence contains 10 words, and E3 protein is the third word in the sentence, then the position of this e3 is 30/100. Finally, the E3 position is transformed into one-hot embedding vector with 100 dimensions.

#### Syntactic information

**Fig. 2:**
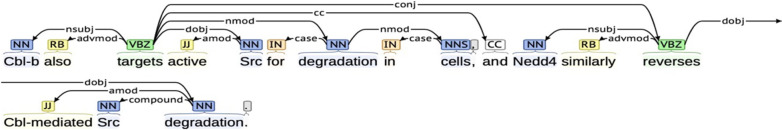
An example for syntactical dependency. Sentence “Cbl-b also targets active Src for degradation in cells, and Nedd4 similarly reverses Cbl-mediated Src degradation.” is parsed by Stanford CoreNLP tool. The parser annotates part-of-speech tags for each word according to their syntactical roles in the sentence, and plots arrow lines for presenting relationship between these words. In this sentence, Cbl-b is E3 ligase, which is the “nsubj” of verb word “targets”

We parsed all sentences, by applying the dependency parser in Stanford Parser tool [11], which is supported by the Application Programming Interface of Natural Language Toolkit (NLTK) in Python and Java Archive. The dependency parser gives a Part-of-Speech (POS) tag to each word in a sentence according to the relationship of all words. Figure 2 shows an example of basic dependencies of one sentence “Cbl-b also targets active Src for degradation in cells, and Nedd4 similarly reverses Cbl-mediated Src degradation.” The dependency tree presents Part-of-Speech (POS) tags of all words, as well as the dependency relationship between them and syntactic structure of the sentence.

After parsing, we scanned for the items that a E3 protein is “nsubj” syntactically associated with a verb word. This pattern could occur anywhere inside the sentence. For example, in Fig. 2, Cbl-b is the E3 ligase with “nsubj” attribute, and “targets” is its associated verb word.

#### Strings information

We extracted string patterns appeared in positive sentences and semantic related with e3-substrate interaction in ubiquitylation in our manual corpus. For example, string “ubiquit” consist of continuous characters in words like “ubiquitylation”, “ubiquitylate”, “ubiquitin” and so on; string “target” consist of continuous characters in words like “targets” and “targeting”. By extracting these key strings that associated with e3-substrate ubiquitylation process, we will obtain more notable feature information.

#### Representation and combination

In order to fully exploit the above semantic-related information, we applied the BioBERT model provided in [12] to represent each information as vector. This is a domain-specific language representation model, which was pre-trained on a large-scale biomedical corpus to solve the problem of ordinary text mining methods cannot handle these medical terms well. After obtain the representation vectors, we applied Principal Component Analysis [13] to adjust each vector dimension to 100.

For each sentence, the above five representation vectors yielded a 500-dimension vector. We applied the L_p_ normalization function on the tensors, each value *v*_*i*_ in the tensor was transformed along one dimension by formula below:

$$v_{i} = \frac{vi}{{{\text{max}}\left( {\left| {\left| {vi} \right|} \right|{\text{p}},{ }\epsilon } \right)}}({\text{x}})$$

Among them, norm applied Euclidean distance calculation, and p was set to 2.

### Deep learning-based classifier

**Fig. 3:**
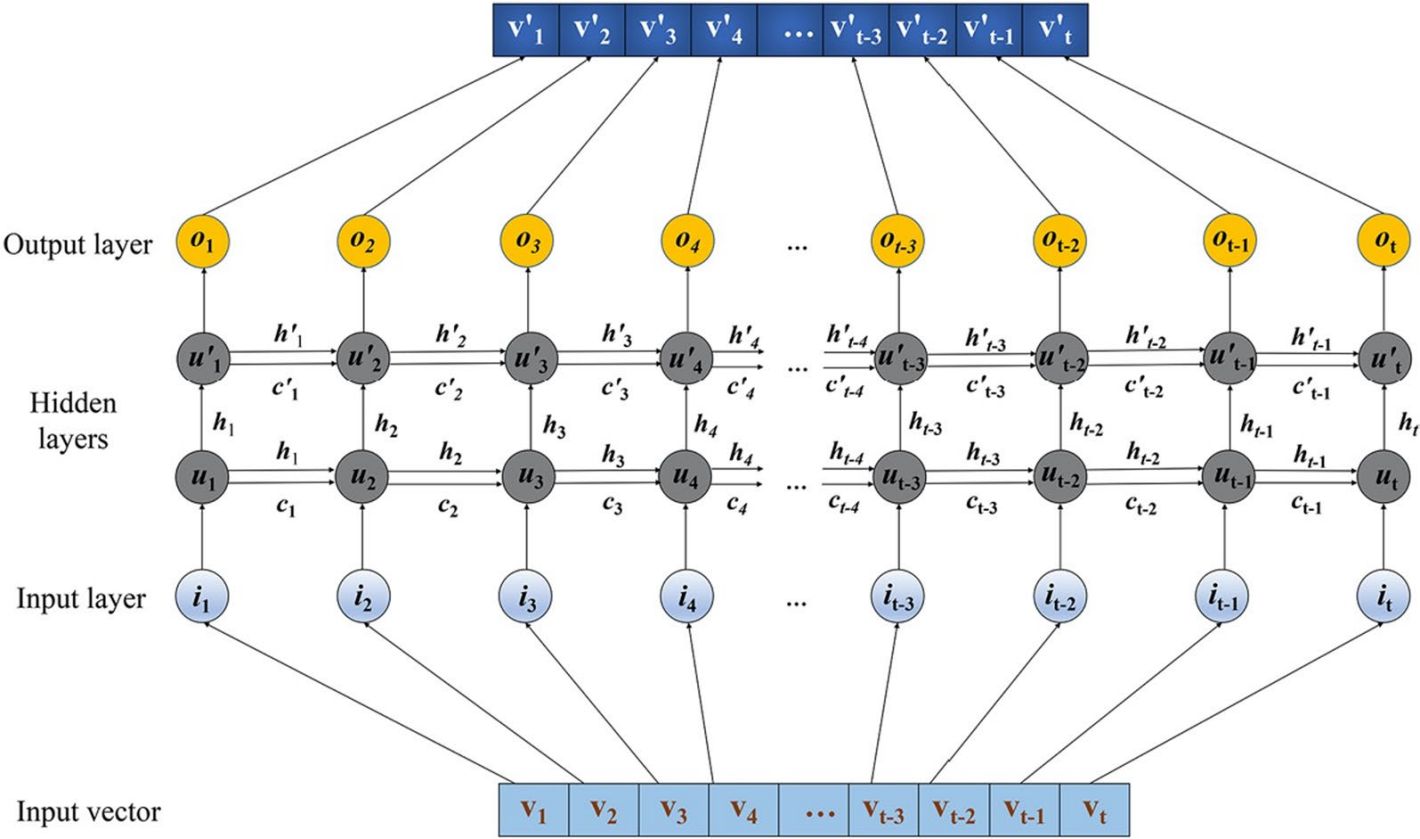
LSTM network in our model. The input is representation vector, and each tensor represents one dimension of vector. Then the tensors are put into hidden layers, number of cells of each hidden layer is set to the size of input tensors. In the hidden layer, each cell would receive information from both previous layer and previous cell

We adopt Long Short-Term Memory (LSTM) deep learning method as the classifier, which firstly handle the input representation vectors. LSTM is one kind of recurrent neural work with attention mechanism, which aims to solve the vanishing gradient problem for Recurrent Neural Networks (RNNs) [14]. In our model, the input are the representation vectors generated in the first stage. Figure 3 details how the LSTM network processes representation vectors, which is the LSTM based encoder layers in Fig. 3.

For the input representation vector (*iv*_1_, *iv*_2_, *iv*_3_, *iv*_4_, …, *iv*_t−3_, *iv*_t−2_, *iv*_t−1_, *iv*_t_) with t = 500 dimensions, each dimension of input vector is regarded as one memory block, and input size is set to t. Then these 500-dimension vectors are processed into the hidden layers, which consists of a serious of self-connected memory cells. In our model, we constructed two hidden layers (u and u′). Hidden size represents the number of cells in each hidden layer, we also set the hidden size as representation vector’s dimension, 500. In the hidden layer, each cell would receive information from both previous layer and previous cell.

For each of the self-connected memory cell, there are three multiplicative units: a input gate, a forget gate and a output gate [14]. Sigmoid function ($\sigma$) with different weights was applied on these gates to assist in hidden state and cell state calculation. These three multiplicative units co-determine information transformation and information storage in the memory cell. Information transformed from previous cell includes a hidden state and a cell state, we initialized both hidden state and cell state to 0, with a tensor shape determined by number of layers = 2 and hidden size = 500.

The hidden state of each cell is calculated below:

$${\text{h}}_{{\text{t}}} = {\text{ o}}_{{\text{t}}} \odot {\text{tanh}}\left( {{\text{c}}_{{\text{t}}} } \right)$$

where h_t_ represents the hidden state at time t, o_t_ represents the output gate, and $\odot$ is the Hadamard product, c_t_ is the cell state which is processed by a tanh function.

The cell state of each cell is calculated below:

$${\text{c}}_{{\text{t}}} = {\text{ f}}_{{\text{t}}} \odot {\text{c}}_{{{\text{t}} - {1}}} + {\text{ i}}_{{\text{t}}} \odot {\text{v}}_{{\text{t}}}$$

where c_t_ represent the cell state at time t, f_t_ represents the forget gate, c_t−1_ represent the cell state at time t−1, i_t_ represents the input gate, and v_t_ is the information from input feature.

We apply the Cross Entropy Loss [15] as the loss function. An optimizer was applied to update parameters according to the gradient descent, and we set the learning rate to 0.001. After the hidden layers, there is a fully connected layer, which transforms the hidden size feature vectors into classes and allocate labels for them. In this layer, we applied a linear transformer activation function. Since this is a binary classification problem, the output number of classes is 2.

### Datasets

#### Data source

Our data comes from our previous work of a database called UbiNet 2.0 [16], which provides 3332 experimentally verified E3-substrate interactions (ESIs) from multiple organisms and total 1560 PMIDs of reference articles. These ESIs came from two sources: experimentally verified ESIs from different databases and ESIs extracted from published literature. It is worth mentioning that for those large mass spectrometry proteomic data sets where many proteins are listed in tables or supplementary data, we made the consideration like this: if the related articles mentioned further experimental verification for these ESIs, we collected them into our database. Also, this database provides different forms of E3 ligases and substrates that collected from related database. For instance, E3 with ID “MDM2_HUMAN” in UniProt, has 6 kinds of expression in the database: “E3 ubiquitylation-protein ligase Mdm2”, “Double minute 2 protein”, “Hdm2”, “Oncoprotein Mdm2”, “RING-type E3 ubiquitylation transferase Mdm2” and “p53-binding protein Mdm2”. It is worth mentioning that this database already contains all the currently known E3 ligases.

#### Data preprocessing

We downloaded the 1560 articles mentioned above, and parse these article texts into sentences. Our goal is to build a model which can identify sentences narrating E3-substrate interaction, and these E3-substrate interaction sentences should contain at least one E3 ligases. Thus, we used the E3 dictionary in UbiNet 2.0 database as filter, to obtain sentences containing as least one E3 ligases.

#### Newly created manual corpus

The filtered sentences containing at least one E3 ligases, while not sure containing substrate(s) or not. In order to obtain E3-substrate interaction sentences data, and also the evaluation data, human judgment is necessary. By annotating labels for each sentence to indicating whether it is a E3-substrate interaction sentence, we could evaluate performance of the model by checking whether it could come up with similar results as could be achieved with human annotation. We randomly selected 3195 sentences as the model training and test sentences for human annotation. We took similar annotation approach as the human created corpus in [17]. Specifically, we invited three volunteers to take part in the annotation. Among them, two performed the first step that manually annotated all the 3195 sentences. In the second step, the third volunteer reviewed the annotation from the first step and unified E3s, substrates (if any) and labels of all the sentences. The sentence labels annotated by the volunteers has been validated by the consistency test. We separate the data in the corpus into training set 2236 (70%) and test set 959 (30%). The data statistics of our corpus and dataset are shown in Table 1.

**Table 1:** Statistics of the datasets

	Positive sentences	Negative sentences	Total
Training	739	1497	2236
Test	317	642	959
Total	1056	2139	3195

## Results

### Evaluation Indictors and Results

We used accuracy, precision, recall, specificity and F1 for measurement. Among them, sentence level accuracy is defined as follow:

1 $${\text{Accuracy }} = (T_{p} + T_{n} )/{\text{N}}$$

Among them, $T_{p}$ represents number of true positive sentences, $T_{n}$ represents number of true negative sentences, N represents the total number of sentences. Precision is defined as follow:

2 $${\text{Precision }} = T_{p} /(T_{p} + F_{p} )$$

Among them, $T_{p}$ represents number of true positive sentences, $F_{p}$ represents number of false positive sentences. Recall is defined as follow:

3 $${\text{Recall }} = T_{p} /(T_{p} + F_{n} )$$

Among, $T_{p}$ represents number of true positive sentences, $F_{n}$ represents number of false negative sentences. Specificity is defined as follow:

4 $${\text{Specificity }} = T_{n} /(T_{n} + F_{p} )$$

Among them, $T_{n}$ represents number of true negative sentences, $F_{p}$ represents number of false positive sentences. F1 is defined based on precision and recall as follow:

5 $${\text{F1 }} = { 2}*{\text{precision}}*{\text{recall }}/ \, \left( {{\text{precision}} + {\text{recall}}} \right)$$

**Fig. 4:**
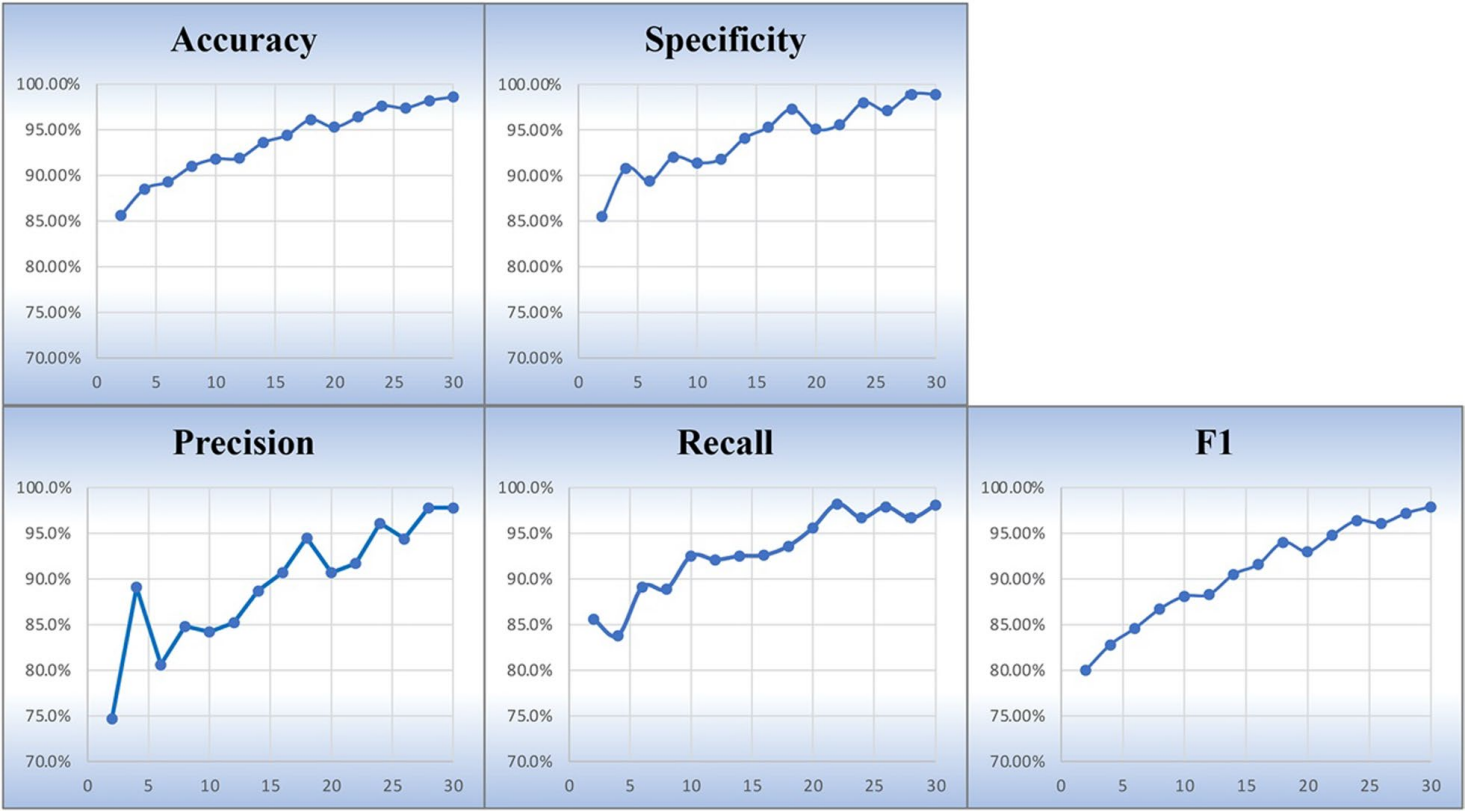
Training results with different values of epoch. X-axis represents value of epoch, and y-axis represents training result on that value of epoch. In general, as the value of epoch increased, result on each indicator increased. These results illustrate that the proposed model in our study has a strong ability to learn information from our corpus, and it is able to achieve high performance on automatic annotation for the E3 ligase-substrate interaction sentences

As mentioned above, we separate our dataset into training data (70%) and test data (30%). The training data and test data were then shuffled into two data loaders in our model, respectively. In neural network, when a complete data set has passed through the network one time and returned once, the process is called an epoch. Number of epochs in the training process can greatly affects the performance of the model. We tested 15 different numbers of epochs range from 2 to 30 with every interval 2 in training stage, and collected results of the model with different epochs. We chose these epoch values because as number of epochs be added, accuracy of training results increased, and it approached to 100% when number of epochs approached to 30. For every value of epoch, we ran the model training program 5 times and collected 5 results on every indicator, and averaged the 5 results as the final result on each indicator for that value of epoch. Figure 4 shows the training performances of model with different epochs on evaluation indicators.

As number of epochs increases, the overall results of model training on each indicator gradually increases with a little fluctuation. When value of epoch approached or reached 30, the results on each indicator tend towards 100%. The precision is 0.747 (lowest) at value of epoch = 2 and then increased, reaching highest value of 0.978 at value of epoch = 30; While the lowest recall 0.838 is at value of epoch = 4, and the highest recall 0.982 is at value of epoch = 22, from value of epoch = 22–30, recall slightly decreased and then increased again. The value of specificity is 0.855 (lowest) at value of epoch = 2, and at value of epoch = 28 and 30 reaches the highest, 0.989; The lowest F1 is 0.800 at value of epoch = 8, and the highest is 0.979 at value of epoch = 30; Besides, accuracy is 0.856 (lowest) at value of epoch = 2 and reaches the highest at value of epoch = 30, which is 0.986. These results illustrate that the proposed model in our study has a strong ability to learn information from our corpus, and it is able to achieve high performance on automatic annotation for the E3 ligase-substrate interaction sentences.

In the meanwhile, we also collected the results of applying the proposed model on test data after training using different numbers of epochs. We found that the test results do not increase absolutely with the increase of epoch value. The test results show that the model has the best performance at value of epoch = 10: precision is 0.728, recall is 0.789, specificity is 0.855, f1 is 0.757 and accuracy is 0.833.

**Fig. 5:**
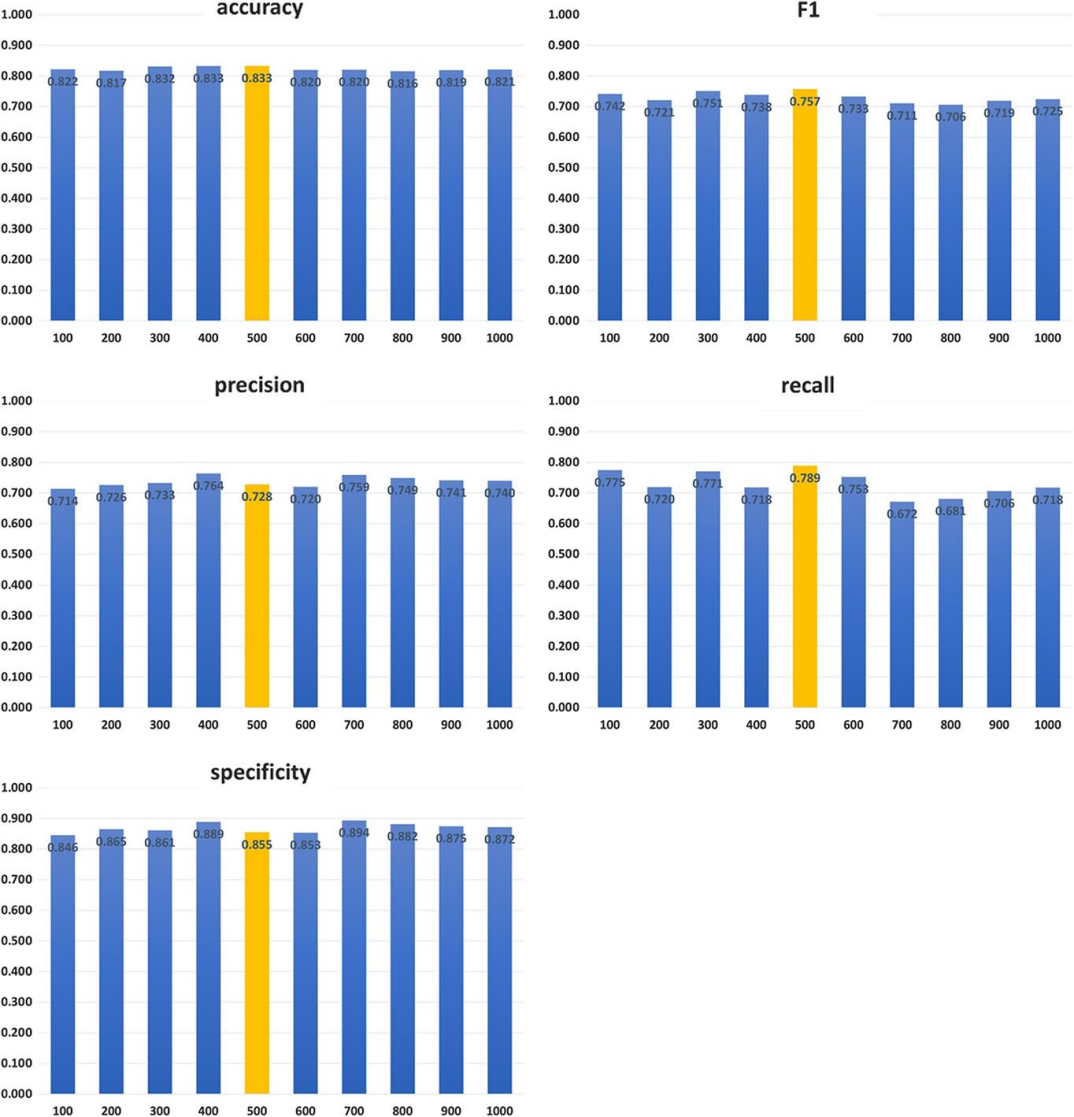
Test results with different dimensions of input vectors. X-axis represents value of dimensions, and y-axis represents test result on that dimension of input vector. Yellow bars are results of model by inputting 500 dimensions vector, which is the value we set for the input vector’s dimension in our proposed model

In order to explore the effective of different dimensions of input vectors on the performance of model, we tested other 9 dimensions of input vectors: 100, 200, 300, 400, 600, 700, 800, 900 and 1,000, to compare model performances on these dimensions of input vectors to model performance on a 500-dimensions vector. Results are showed in Fig. 5.

Although there is not much a difference between results of different dimensions of input vectors on each evaluation indicator, in terms of accuracy and F1, model with 500 dimensions input vector has the best performance.

### Comparison with other machine learning based classifiers

We also applied statistical machine learning algorithms: Support vector machines, linear logistic regression, decision trees and random forests, and a deep learning method without any attention mechanism as classifiers for comparison. Representation stage and the input representation values are the same with our proposed model.

#### Support vector classifier

The support vector machine (SVM) is a supervised linear machine learning model, which is the most classical and effective method for binary classification and can be well applied in text classification [18–20]. We used the “Radial Basis Function” kernel, and set the class weight as “balanced” since it would automatically adjust weights of each class according to their frequencies in the input data.

#### Logistic regression

Logistic Regression is a model in statistical science, which in its basic form uses a function to construct a binary dependent variable classifier, and the independent variables are a set of (influential) factors [21]. From a mathematical point of view, a binary model has a dependent variable with two possible values, e.g., forward/backward, which can be labeled as "0" and "1", where the corresponding probability values of labels are in [0, 1]. The output probability values are linearly related to independent variables in the model. Also, the dependent variables can be of multiple classes. Logistic Regression is widely used in text mining for binary classification.

#### Random forest

A random forest is composed of multiple decision trees whose output is determined by the output of each decision tree. Decision trees are tools that take known independent variables (attributes) and probabilities of occurrence (outcomes) of dependent variables and put them into a tree structure to support classification by constructing nodes and branches of the decision tree [22]. In our model, we set number of tress in the forest to 100, set split criterion to “gini”, and bootstrap samples were also used.

#### Neural network

This is a supervised learning-based classifier. There could be arbitrary number of hidden layers in the neural. Given the input features with n dimension vectors {v_1_, v_2_, v_3_, …, v_n_}, the input layers would contain n neuron to these vectors. And then each neuron would process the received vector with an activation function, and transform information to next corresponding neuron in the next hidden layer. The output layer would transform information from last hidden layer into probability value of each class. In this neural network classifier, we applied 150 hidden layers, and the activation function is set to “Rectified Linear Unit”.

### Comparison results

To obtain performance of each method, we applied 10 cross validation strategy in training process. That is, splitting training data into 10 consecutive folds with same amount of data in each fold, and without any shuffling. Then used 9 of the folds as training data and the remained fold as test data, repeated this procedure 10 times until all folds has been used as test data one time, finally obtained 10 results and averaged them. We collected results of all traditional machine learning based methods on each indicator. Results are showed in Table 2.

**Table 2:** Performances of machine learning based models on training data

Classifier	Precision	Recall	F1	Accuracy	ROC_AUC
SVM	0.67	0.89	0.76	0.80	0.88
Logistic regression	0.68	0.86	0.75	0.79	0.88
Random forest	0.75	0.72	0.73	0.81	0.89
Neural networks	0.74	0.74	0.74	0.82	0.89

Training results show that for F1, accuracy and Area Under Curve (AUC) values, results of four methods do not do not differ significantly: among them, difference in F1 is within 0.03, difference in accuracy is also within 0.03, and difference in AUC is only within 0.01. SVM and logistic regression methods outperformed other two methods in terms of F1 scores (0.76 and 0.75), deep learning neural networks and random forest are slightly inferior but not bad, with F1 values of 0.74 and 0.72. Beside, neural networks obtains the highest accuracy (0.82) among all methods.

The training process and results are based on the training data in our corpus. To verify the performance of traditional machine learning based methods, we also applied these models using the test data from corpus. In other words, the model was trained using 70% of the data in our corpus, and then the trained model was applied to the remaining 30% test data to collect results. Test results are showed in Table 3.

**Table 3:** Performance of machine learning based models on test results

Classifier	Precision	Recall	Specificity	F1	Accuracy
SVM	0.614	0.870	0.731	0.720	0.777
Logistic regression	0.635	0.826	0.766	0.718	0.786
Random forest	0.719	0.712	0.863	0.715	0.813
Neural networks	0.728	0.703	0.871	0.715	0.815

SVM obtained the highest F1 (0.720) among these methods; Second is logistic regression, with F1 value of 0.718; and performance of random forest and neural networks are comparable, which have a negligible difference from the first two methods. In terms of accuracy, the neural networks and random forest methods outperform the first two, with accuracy values of 0.815 and 0.813, versus accuracy values of 0.777 and 0.786. However, comparing with random forest and neural networks, the performance of SVM and logistic regression in terms of precision is poor, with values of 0.614 and 0.635, while the other two have precision scores of 0.719 and 0.728. And for recall scores, the former two methods outperform the latter two methods.

**Fig. 6:**
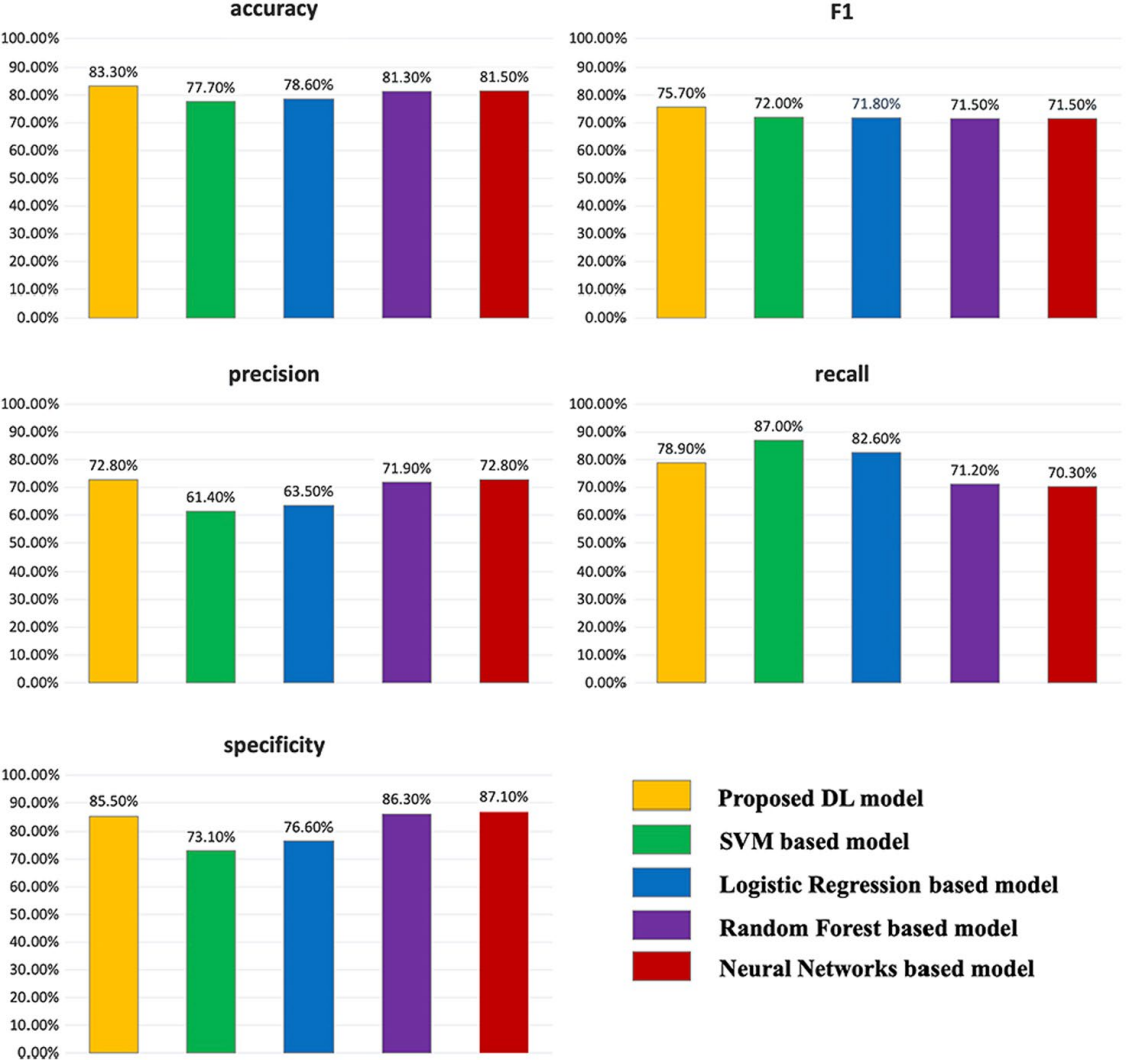
Performance comparison for different models

Although the models constructed using these machine learning-based methods have achieved well performance on each indicator and already fulfilled the requirement of E3 ligase-substrate interaction sentences annotation. In comparison, our proposed deep learning-based model performed better. To analyze the performance of the models based on each method, we draw the comparison figures of the test results over all methods (in Fig. 6).

As can be seen from the figure, in general, our proposed deep learning-based model has the best performance since it obtained the highest scores on both f1 and accuracy indicators. Although SVM and logistic regression obtained higher recall scores than other methods, their performances on precision scores are poor. By comparison, random forest and neural networks obtained balancing results on precision and recall, but our proposed model performed the best.

## Discussion

The advantage of representation is that it can effectively obtain the semantic relationship between e3 ligase and sentence, thus helping the model to make better judgments. The well representation also made the rest of tasks in this research simpler and well fit with the attention based deep learning used subsequently. We applied the LSTM network for classification and model construction, which is an attention based RNNs deep learning method. It has the advantage of ameliorating long-term dependency problem in RNNs by applying a gated mechanism to select valid information to be put into cell state and passed to the next cell or the next layer. In addition, as a nonlinear model, LSTM can also be used as a complex nonlinear unit for constructing larger deep neural networks.

Our experimental results showed that the proposed model performed well on both training data and test data, which proves the effectiveness of the model and affirms our methods. Comparing with other machine learning based models, our proposed model obtained the best performance, affirming the significant positive effect of attention based deep learning method on E3-substrate interaction sentences annotation. In general, all of the four comparison methods are fully capable of handling the issue of E3 ligase-substrate interaction sentences annotation in our study. Among the other methods, SVM as the most popular and was considered the best method for binary classification problem, also performs the best in solving issue in our study, with f1 achieved the highest among these methods. Followed by another binary classification method, logistic regression. Since the task of annotating sentences in this research is also a binary classification task: the sentences are divided into those that narrate E3-substrate interaction and those that do not state this, it is reasonable that these two methods perform better from an overall perspective. However, the performance of SVM and logistic regression in terms of precision is poor, but they obtained higher recall scores—this indicates that the model annotation results have a high number of false positive, which proves that these two methods are weak in balancing the fitting parameters. While the deep learning neural network and random forest perform better from this aspect, with balanced precision and recall values, which was demonstrated in both training and test procedure, affirms their ability of parameter adjustment. Neural network is a deep learning method, got accuracy score higher than other three methods. But it does not contain any attention mechanism inside the network as LSTM, because of excessive gradient changed with increasing number of network layers, it presented a mediocre performance in terms of F1 score. By comparison, the attention mechanism in LSTM helps with vanishing gradient problem, it can improve gradient flow. As for the proposed model in our research, the attention mechanism can better integrate and filter the information from representation. Thus, the performance of our proposed attention based deep learning model is better than other comparison models.

As we have a complete database for E3 proteins, we applied this database in data preprocessing as E3 dictionary, to filtered related sentences. Thus, in fact, the task of the model is to select the sentences that describe E3-substrate interaction from the sentences that containing E3 ligases. In reality, this method relies on E3 information, even most of E3 proteins are already known. In future, we propose to develop model that do not rely on any independent E3 information.

## Conclusion

In this research, we proposed a model based on representation and attention based deep learning methods, to automatic annotate E3-substrate interaction sentences in biomedical literature. Focusing on the sentences with E3 protein inside, we applied natural language processing techniques to generate representation vectors. Then we input the vectors into a LSTM-based deep learning classifier for model training. Experimental results had proved the effectiveness of our proposed model. Besides, we also compare our model with other statistical machine learning methods, experimental results showed that our model achieved the best performance among all the methods.

To the best of our knowledge, there is no relevant study focusing on automatic annotation of E3 ligase-substrate interaction information from literature. The significance and contribution of this research are twofold: first, we created a manual corpus of E3-substrate interaction sentences, in which the E3 proteins and substrate proteins are also labeled; second, we built a model to automatic annotate E3-substrate interaction sentences through natural language processing based representation and attention based deep learning methods. From biomedical perspective, this research can assist in further screening of key ubiquitylation ligase substrates for in-depth studies, in the meanwhile, promote the understanding of the cellular regulatory processes involved in ubiquitylation. And also, it can help to reveal the role of E3 ligase-substrate interactions in cancer occurrence and development in cancer cells, and understand the pathogenesis of ubiquitylation-related cancers, which is important for the development of highly specific and low side effect drugs.

## Data Availability

The data-sets used and/or analysed during the current study available from the corresponding author on reasonable request.
